# Formation and photochemical investigation of brown carbon by hydroxyacetone reactions with glycine and ammonium sulfate†

**DOI:** 10.1039/c8ra02019a

**Published:** 2018-06-06

**Authors:** Yan Gao, Yunhong Zhang

**Affiliations:** School of Chemistry and Chemical Engineering, Beijing Institute of Technology Beijing 100081 China yhz@bit.edu.cn; School of Materials and Chemical Engineering, Bengbu College Bengbu 233030 China 2857965284@qq.com

## Abstract

Increasing attention has been paid to atmospheric “brown carbon” (BrC) aerosols due to their effect on the earth's climate. Aqueous BrC aerosols were formed through aqueous reactions of hydroxyacetone (HA) with nitrogen compounds such as glycine (Gly) and/or ammonium sulfate (AS). When exposed to nitrogen compounds for several days, HA, as a type of aqueous carbonyl compound, becomes absorbent and fluorescent in the blue visible and near ultraviolet regions, which have been monitored by UV/vis and fluorescence spectroscopy. In this study, we quantified absorption and excitation-emission matrix (EEM) spectra in the formation of aqueous BrCs, which was generated from HA-Gly and HA-Gly-AS mixtures, respectively. The obtained data was used to determine the base-10 absorption coefficient (*α*), absorption Angstrom exponent (AAE), and effective quantum yield (QY). All of the related parameters provide further evidence for the formation of aqueous BrC. The absorbances of the as-obtained BrCs follow the order HA-Gly-AS > HA-Gly > HA-AS. In other words, HA-Gly-AS mixtures displayed the most intense absorbances, whereas HA-AS mixtures barely produced visible absorbance. It is reasonable to speculate that Gly promotes the formation of HA-Gly BrC mixtures. The experimental results are consistent with previous measurements reported by Powelson *et al.* BrCs from HA-Gly-AS and HA-Gly exhibit stronger fluorescence between 300 and 400 nm. Glycine plays a more important role in the formation of aqueous BrC than that of AS. Furthermore, we examined the mass absorption coefficient (MAC) by photolysis of aqueous BrCs, which resulted from the oxidation of HA-Gly and HA-Gly-AS mixtures by 5 mM H_2_O_2_. An effective photolysis time induced significant changes near-UV (300–400 nm) absorption intensity of HA-Gly and HA-Gly-AS mixtures. These results emphasize the dynamic nature of the corresponding atmospheric aqueous BrC. Overall, our study provides the optical properties of the corresponding atmospheric aqueous BrC and the H_2_O_2_ oxidation photolysis process of the as-obtained BrC in detail, which may contribute to the understanding of the important effects of aqueous BrC for atmospheric chemistry and climate.

## Abstract 2

Increasing attention has been paid to atmospheric “brown carbon” (BrC) aerosols due to their effect on the earth's climate.

## Graphical abstract

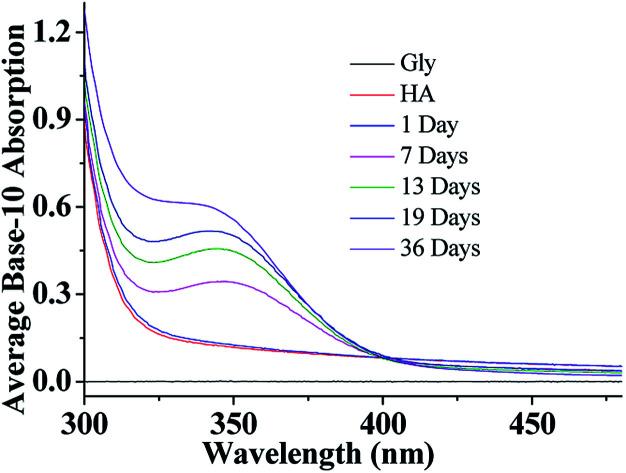

## Introduction

Atmospheric aerosols play a key role in modulating the Earth's radiative balance, particularly positive radiative forcing (warming) and the average surface temperatures on both global and local scales by direct and indirect mechanisms.^2–4^ They have also been linked to a significant adverse impact on public health, particularly in the occupational environmental effects by airborne dispersal and transport.^5–7^ Atmospheric “brown carbon” (BrC), as a collective term for light absorbing but poorly characterized organic compounds in the atmosphere, is an important contributor to light absorption and climate forcing.^8–10^ As a category of condensed-phase organic carbonaceous compounds that are present in atmospheric aerosol from both anthropogenic and natural sources, BrC can absorb solar radiation in the blue visible region and near ultraviolet region with strong wavelength dependency; the light absorption of this type of absorbent organic matter in the ultraviolet band increases as the wavelength becomes shorter.^11–14^ The sources and compositions of BrC are very complex, and we can generalize the source into two major categories: important primary emissions from biomass burning and the incomplete combustion discharge of biofuels. In addition, there is secondary emission from secondary organic aerosols (SOA) formed from atmospheric secondary transformation and aging of atmospheric particulate matter from fresh or secondary conversion.^15–17^ However, emission reductions are possible only when the sources, optical properties and chemical compositions are identified.^1^ In polluted areas, many organic carbonaceous compounds that are routinely found in clouds and aerosol have the potential to form fluorescent and absorbent BrC products.^18^

Fluorescence spectroscopy that can detect and record emission spectra in real time is a rapid and sensitive technique for quantitative and qualitative analysis of BrC.^19–21^ Fluorescent particles must contain significant amounts of organic compounds with large absorption coefficients and fluorescence quantum yields.^22^ The small, water-soluble carbonyl compound hydroxyacetone (HA) can undergo browning (Maillard) reactions in the presence of ammonium salts or amino acids such as Gly.^23–32^ Comparable to (or slightly longer than) the average lifetime of aerosol particles in the troposphere, the maximum reaction time of the aqueous BrC solutions used in the research is up to 19 days.^33^

In the present study, aqueous BrC aerosols were formed through the aqueous reactions of hydroxyacetone (HA) with nitrogen compounds such as glycine (Gly) and/or ammonium sulfate (AS). We provide further evidence for the formation of BrC using the related parameters of excitation-emission matrix (EEM) fluorescence spectra, fluorescence emission spectra and absorption spectra, such as wavelength-independent fluorescence quantum yield (QY), Stokes shift, wavelength-dependent MAC, and absorption Angstrom exponent (AAE). We preliminarily identified the formation process of BrCs from HA-Gly-AS and HA-Gly mixtures. Furthermore, we studied a series of photochemical processes of the as-obtained BrC after H_2_O_2_ oxidation photolysis at the laboratory scale.

## Experimental section

Aqueous stock solutions were prepared in deionized water using ammonium sulfate (>99.0%, Beijing Chemical Works), glycine (>99.0%, Tianjin Guangfu Fine Chemical Research Institute) and hydroxyacetone (90%, Aladdin Industrial Corporation). BrC stock solution from HA-Gly mixtures was prepared by mixing 15 mL of glycine (0.6 M), 15 mL of hydroxyacetone (0.6 M), and 15 mL of ultrapure water. The final concentrations in the stock solution were ~0.2 M for glycine and ~0.2 M for hydroxyacetone. BrC stock solution from HA-AS mixtures was prepared by mixing 15 mL of ammonium sulfate (0.6 M), 15 mL of hydroxyacetone (0.6 M), and 15 mL of ultrapure water. BrC stock solution from HA-Gly-AS mixtures was prepared by mixing 15 mL of ammonium sulfate (0.6 M), 15 mL of hydroxyacetone (0.6 M), and 15 mL of glycine (0.6 M). BrC stock solutions were prepared using a method similar to that developed previously in order to simulate atmospheric aerosol conditions, which are known to be inset in these systems previously.^34^

A U-3900 UV/Vis spectrophotometer (Hitachi, Japan) at a scan rate of 300 nm min^−1^ was employed to record changes in absorbance between 300–480 nm in a 1 cm-quartz cell. A reference absorption spectrum using deionized water as the background was determined in the same quartz cell. An F-97 fluorescence spectrometer (Pgeneral, Shanghai, China) was used in EEM mode (0.01 s response, high sensitivity, 600 nm min^−1^ emission scan speed, 10 nm data pitch) to map the development of fluorescence over the entire range of excitation (*λ*_ex_) and emission wavelengths (*λ*_em_) (typically 280–450 nm) with the limit *λ*_em_ = (*λ*_ex_ + 20 nm) to avoid the Rayleigh and Raman scattering signal. In brief, for the EEM spectra, the excitation wavelength varied over the 290–450 nm range in 10 nm steps, and the emitted fluorescence strength was recorded over the 310–600 nm range in 1 nm steps. In H_2_O_2_ oxidation photolysis of BrC solutions, 1.49 mL 0.20 M BrC stock solution and 0.01 mL 0.75 M hydrogen peroxide (30%, Wuxi City Yasheng Chemical CO., LTD) were mixed thoroughly, and the solutions were placed in a 1 cm quartz cuvette for 254 nm illumination (Ultraviolet Analyzer) and absorption measurements. FTIR measurements were carried out using a Thermo Scientific Nicolet is 10 spectrometer. Samples were analyzed at ambient temperature with a scan range of 400–4000 cm^−1^, at a resolution of 0.4 cm^−1^.

## Results and discussion

### UV/Vis light absorption

**Fig. 1:**
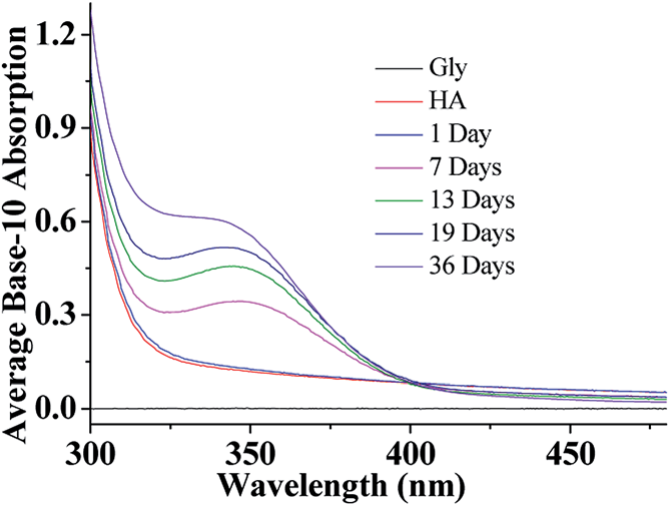
UV/Vis absorption spectra of HA-Gly mixtures as a function of reaction time from 300–480 nm.

After a selected reaction period (1–19 days), UV/Vis absorption measurements of the aqueous HA-Gly mixtures were carried out over the wavelength range of 300–480 nm as a function of reaction time using a U-3900 UV/Vis spectrophotometer (Fig. 1). The base-10 absorption coefficient *α* of the BrC solutions was calculated by dividing the base-10 absorption strength by the quartz cell path length, *α*_SOA_(*λ*) = *A*(*λ*)/l.^22^ The background was determined by measuring the absorption of deionized water.

As shown in Fig. 1, compared to Gly and HA, the absorption of aqueous HA-Gly mixtures significantly enhanced with reaction time in the blue visible and near ultraviolet regions. It should be noted that absorption at longer wavelengths (>480 nm) also increases with reaction time (36 days), but the absolute changes are too small to visualize. The spectral data show that the mixtures of HA-Gly display broad absorption bands peaking near 350 nm.

A power law was used to describe the absorption Angstrom exponent (AAE) of BrC:1
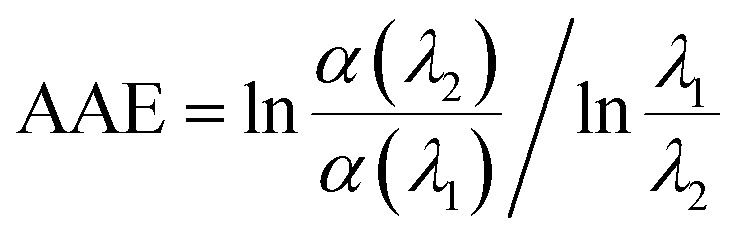
where AAE is the measure of the spectral dependence of aerosol light absorption, which is required for determining the absorption coefficient that is obtained at a limited range of wavelengths.^35^ Values of AAE in excess of 1, which are typical for a BC dominated aerosol, indicate a strong contribution of BrC to aerosol absorption.^36^ The AAE of the HA-Gly mixtures studied herein is approximately 5 (Table 1) for the visible wavelength range of 400–480 nm, overlapping the AAE range of atmospheric and water-soluble BrC. In summary, the HA-Gly mixtures can be regarded as an example of a moderately-absorbing type of BrC.

**Table 1:** Measured base-10 absorption coefficients *α* correspond to a 0.058 M QS solution and 0.20 M HA-Gly mixtures. The integrated emission intensities, *I*_area_, correspond to the 350 nm excitation from 362 nm to 600 nm. The effective fluorescence quantum yield (QY) values were calculated from eqn (2). The last two columns contain the fluorescence index (FI) and the index of recent autochthonous contribution (BIX)

Solution	*α* (350 nm) cm^−1^	*I*_area_	QY%		AAE 400–480 nm	FI	BIX
QS (5.8 × 10^−7^ M)	0.00603	12 269.4	51		—	—	—
HA-Gly (7 days)	0.34	20 389.6	1.50		4.29	2.11	0.75
HA-Gly (13 days)	0.45	22 083.9	1.23		5.13	2.13	0.70
HA-Gly (19 days)	0.50	22 392.0	1.12		4.88	2.13	0.72
HA-Gly (36 days), before photolysis	0.56	23 239.1	2.04	7.60		2.12	0.68
HA-Gly (36 days), after photolysis	0.11	11 049.2	2.52	5.18		2.19	0.82

Similarly, compared to HA-Gly mixtures, the absorption of aqueous HA-Gly-AS mixtures was also significantly enhanced with reaction time in the blue visible and near ultraviolet regions (Fig. 1S(a)†). The AAE for the HA-Gly-AS mixtures studied herein range from 9 to 14 (Table S1†) for the visible wavelength range of 400–480 nm. Hence, the HA-Gly mixtures also can be regarded as an example of a moderately-absorbing type of BrC. However, for the HA-AS mixtures, absorption enhancement can barely be observed over the entire spectral range with reaction time, as shown in Fig. S1(b).† Above all, we can conclude that the HA-AS mixtures cannot be considered as a type of BrC.

### Excitation-emission spectra and effective quantum yield

#### Excitation-emission matrix fluorescence spectra

**Fig. 2:**
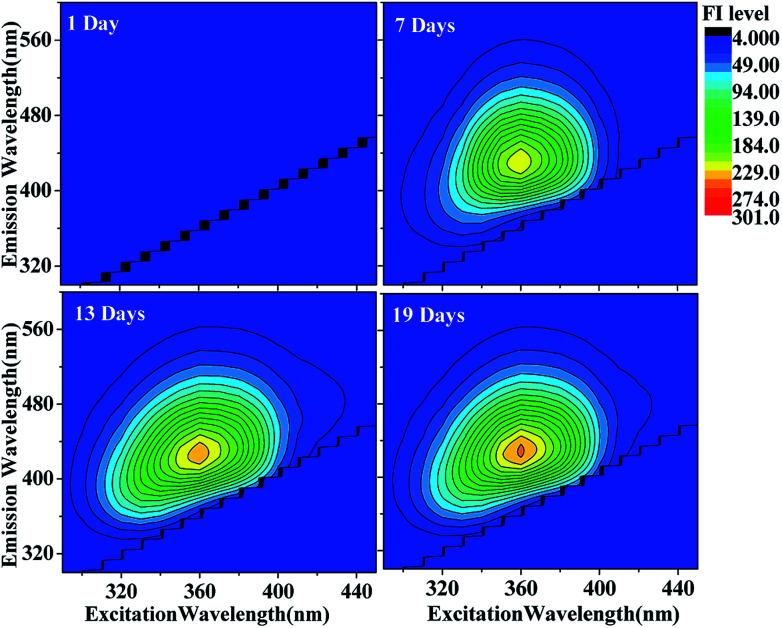
EEM plots of HA-Gly mixtures as a function of reaction time. The fluorescence intensity (FI level, color coded as shown on the right) increased somewhat as a function of reaction time.

Excitation-emission spectra are commonly presented in the form of a three-dimensional excitation-emission matrix (EEM) plot, wherein the emission intensity is displayed in a contour plot as a function of excitation and emission wavelengths.^37,38^ The EEM plots for the HA-Gly mixtures as a function of reaction time were similar with similar contour patterns, and the only difference was that their relative emission intensities increased slightly, while the peak shape and position did not change significantly, as shown in Fig. 2. Because of the insufficiently strong emission from the HA-Gly mixtures, we opted to remove the stronger Rayleigh and Raman scattering peaks from the EEM plot. The HA-Gly mixtures in three different periods exhibited maximum emission intensities when they were excited at around 360 nm, and the average Stokes shift, defined as the average difference between *λ*^max^_em_ and *λ*_ex_, was about 77 nm. However, compared to the HA-Gly mixtures, the emission intensities of the HA-Gly-AS mixtures were considerably stronger, as shown in Fig. 2S.† However, the HA-Gly-AS mixtures exhibited maximum emission intensities when they were excited at around 360 nm, and the average Stokes shift was about 77 nm, which was similar to that for the HA-Gly mixtures.

The EEM intensities at different excitation and emission wavelengths can be used as indicators of the type and source of the dissolved organic matter such as terrestrially derived fulvic acids and fulvic acids of microbial origin in fog water.^22,41^ The following indicators determined: fluorescence index (FI) was calculated using the ratio of emission intensities at 450 nm and 500 nm following 370 nm excitation,^39^ and the index of recent autochthonous (*i.e.*, produced by the original source of dissolved organic matter as opposed to externally produced^22^) contribution (BIX) was calculated using the ratio of emission intensities at 380 and 430 nm following 310 nm excitation.^40^Table 1 lists both indices calculated for the HA-Gly mixtures probed in this study. In general, FI values of approximately 1.4 are observed for terrestrially derived fulvic acids, while FI values of approximately 1.9 corresponds to fulvic acids of microbial origin.^41^ BIX values of approximately 0.6 corresponds to dissolved organic matter with low autochthonous component, while BIX > 1 is observed for dissolved organic matter of biological origin.^22,42^ In our study, the observed FI values of the HA-Gly mixtures is 2.1 (Table 1), which is close to 1.9 according to the definition of FI. Hence, the HA-Gly mixtures probably correspond to fulvic acids of microbial origin. The BIX values for the HA-Gly mixtures are on average 0.7 (Table 1), which is close to 0.6 and consistent with a low autochthonous component. In other words, the reaction might have been generated from biological origin fulvic acids of microbial origin but the concentration is very low.^42^ Similarly, we calculated the FI value of 2.1 and BIX value of 0.6 (Table S1†) for the HA-Gly-AS mixtures, which were also similar to that of the HA-Gly mixtures.

#### Effective quantum yields

The effective quantum yields (QY) were measured as described by Lee *et al.*^22^ using quinine sulfate (QS, (C_2_0H_24_N_2_O_2_)_2_·H_2_SO_4_·2H_2_O) dissolved in 0.05 M sulfuric acid as a reference compound. QS is a convenient standard and QY_QS_ = 51%.^43,44^ The background for the spectrum was 0.05 M sulfuric acid for the reference compound measurements because the solution didn't exhibit any detectable absorption and fluorescence. The absorption spectrum shows QS peaks at 350 nm (Fig. S7(a)†), and this excitation wavelength was chosen for the QS fluorescence measurements, as shown in Fig. S8(a).† The corresponding absorption coefficient calculated from the linear fit in Fig. S7(b)† is 0.00603 cm^−1^ at the peak of the QS absorption spectrum (350 nm) using 5.8 × 10^−7^ M QS solutions.

The corresponding integral area between 362 and 600 nm calculated from the linear fit in Fig. S8(b)† is 12 269.4 using 5.8 × 10^−7^ M QS solutions. The QY values of the BrC solutions in three different time periods were calculated at 350 nm. excitation wavelength by comparing the measured fluorescence emission intensities from the EEM plots integrated over the emission wavelengths (*I*) and the base-10 absorption coefficients (*α*):^22^2
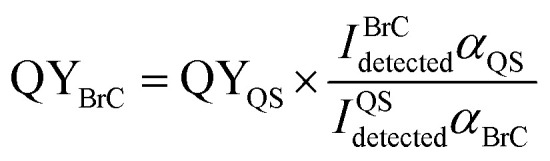


The resulting QY values of the HA-Gly mixtures are summarized in Table 1. Fig. 1 demonstrates that the absorption coefficients of the HA-Gly mixtures increase significantly in different time periods. However, the effective fluorescence QY values decrease slightly from 1.50% to 1.12%, and they have a same order of magnitude (~0.01). Meanwhile, the effective fluorescence QY values of the HA-Gly-AS mixtures were also calculated, and they are in the range of 2.56–1.57% (Table S1†), which are higher than that of HA-Gly mixtures. These results probably illustrate that Gly are more important in BrC formation than AS, and Gly may have positively contributed to the formation of BrC.

### H_2_O_2_ oxidation photolysis of BrC solutions

#### Mass absorption coefficients of BrC solutions

**Fig. 3:**
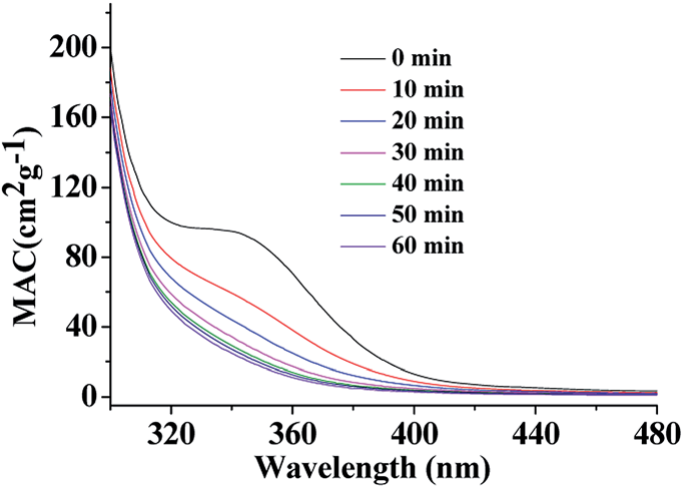
UV/Vis absorption spectra recorded during photolysis of HA-Gly mixtures in the 300–480 nm range. The vertical axis corresponds to the mass absorption coefficient (MAC) calculated from eqn (3).

Aqueous brown carbon species are organic components and susceptible to photochemical degradation and their optical properties are altered. Hence, the H_2_O_2_ oxidation photolysis experiments of HA-Gly mixtures were conducted and displayed in Fig. 3. The importance of the steady state concentration of H_2_O_2_ radicals is evident in the H_2_O_2_ oxidation experiments.

UV/Vis absorption spectra were recorded using a UV-Vis spectrometer with pure solvent as a reference. The extent of HA-Gly mixtures browning was quantified in terms of the effective mass absorption coefficient (MAC) of the organic material. Wavelength-dependent MAC (cm^2^ g^−1^) can be directly calculated from the base-10 absorbance, *A*_10_, of the HA-Gly mixtures with known mass concentration, *C*_mass_ (g cm^−3^), and path length, *b* (cm).^35^3
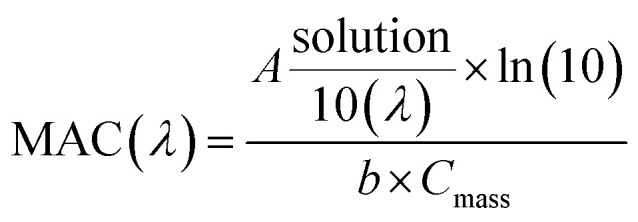


The MAC spectra of HA-Gly mixtures before and after H_2_O_2_ oxidation taken at different photolysis times are shown in Fig. 3. The shapes of the spectra agree with the characteristics of a typical atmospheric BrC material.^45^ The absorption coefficient is the highest in the near-UV range, and there is a tail in the visible range. In contrast, the H_2_O_2_ oxidation photolysis experiments of HA-Gly-AS mixtures were conducted (Fig. S3†). The experimental results are similar to that of HA-Gly mixtures.

#### Effective rate of aqueous BrC solution photolysis

**Fig. 4:**
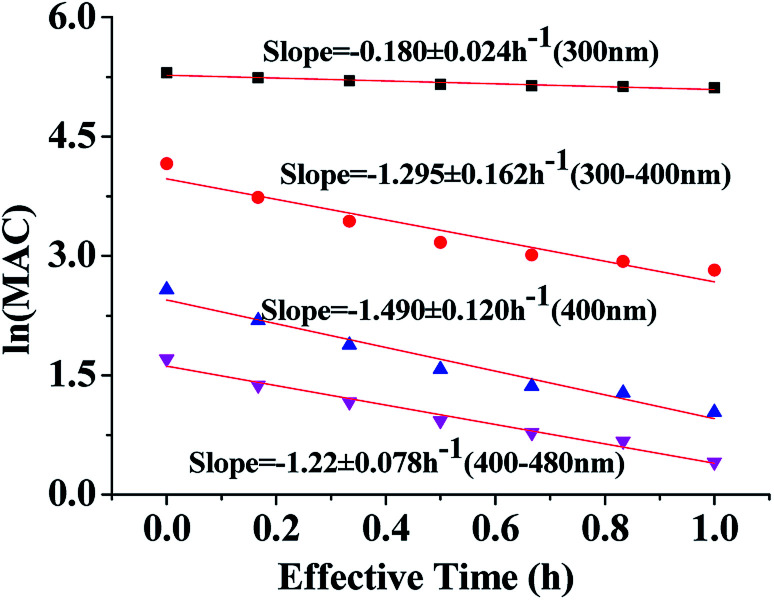
Decay of absorbance during irradiation of HA-Gly aqueous mixtures. The average ln(MAC) is plotted against the effective photolysis time at 300 nm (black square), in the near-UV region (300–400 nm, red roundness), at 400 nm (blue regular triangle) and in the visible region (400–480 nm, pink inverted triangle).

As shown in Fig. 4, the exposure of aqueous HA-Gly mixtures to an ultraviolet analyzer moderately reduces its MAC, supposedly because of photodegradation of the chromophores of the HA-Gly mixtures. Although they have been omitted from Fig. 3, the MAC data continues to decline at the same rate with increasing photolysis time. Because of the very large number of individual chromophores in the HA-Gly aqueous mixtures, quantitative interpretation of these measurements is rather complicated.

The effective rate constant *k*, considering the first-order kinetic process, is commonly used to approximate the MAC decay.^36^4
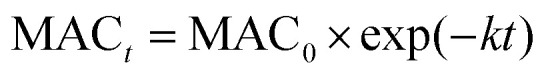


Fig. 4 shows the dependence of the average MAC of HA-Gly solutions at 300 nm, in the near-UV regions (300–400 nm), at 400 nm, and in the near-visible regions (400–480 nm), on the effective H_2_O_2_ oxidation photolysis time. The effective rate constant *k* followed the order 400 nm (*k* = 1.490 h^−1^) > the near-UV range (*k* = 1.295 h^−1^) > the visible range (*k* = 1.218 h^−1^) > 300 nm (*k* = 0.180 h^−1^).

These effective *k* values can be converted into empirical half-lives, *τ* = ln(2)/*k*^36^ which are listed in Table 2. We also include data on photodegradation of HA-Gly mixtures produced by the reaction of H_2_O_2_ oxidation. The distinctive peak at approximately 340 nm almost completely disappears after 1.0 h of photolysis, and the brown color of the solutions became shallow in the process. Fig. S4† shows the dependence of the average MAC of HA-Gly-AS mixtures on the effective H_2_O_2_ oxidation photolysis time. The effective rate constant *k* followed the order 400 nm (*k* = 1.54 h^−1^) > the visible range (*k* = 1.364 h^−1^) > the near-UV range (*k* = 1.242 h^−1^) > 300 nm (*k* = 0.226 h^−1^).

**Table 2:** Effective rate constant *k* and effective half-life *τ* (in h) for the disappearance of absorbance at different wavelengths in HA-Gly mixtures

Wavelength (nm)	300	Near-UV	400	Visible
*k*	0.180	1.295	1.490	1.218
*τ*	3.851	0.535	0.465	0.569

#### Fluorescence of BrC solutions

**Fig. 5:**
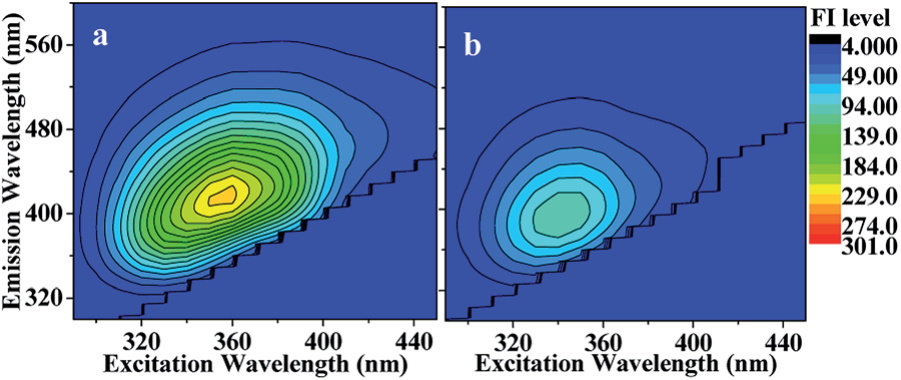
EEM spectrum recorded (a) before and (b) after photolysis of HA-Gly mixtures. After 60 min of photolysis, the fluorescence intensity (FI level, color coded as shown on the right) decreased somewhat (meanwhile the solution absorbance decreased, as shown Fig. 4).

The EEM spectra in Fig. 5 shows that HA-Gly mixtures were moderately fluorescent both before and after photolysis. The fluorescence quantum yield (QY) of the HA-Gly mixtures, measured as described in eqn (2), increased from 2.04% to 2.52%. The H_2_O_2_ oxidation photolysis reduced the overall absorption coefficient of the HA-Gly mixtures (Fig. 3), and there was a significant decrease in their fluorescence intensity, as shown in Fig. 5b. This is an expected result considering that H_2_O_2_ oxidation photolysis probably reduced the average number of double bonds, thus preferentially removing highly unsaturated molecules that are more likely to fluoresce. Fig. S5† in the ESI† shows a typical EEM spectrum for the HA-Gly-AS mixtures before and after H_2_O_2_ oxidation photolysis. The QY, described in Table S1,† increased from 1.11% to 2.15%, and the fluorescence intensities also decreased after H_2_O_2_ oxidation photolysis in Fig. S5(b).†

#### FTIR spectral properties

**Fig. 6:**
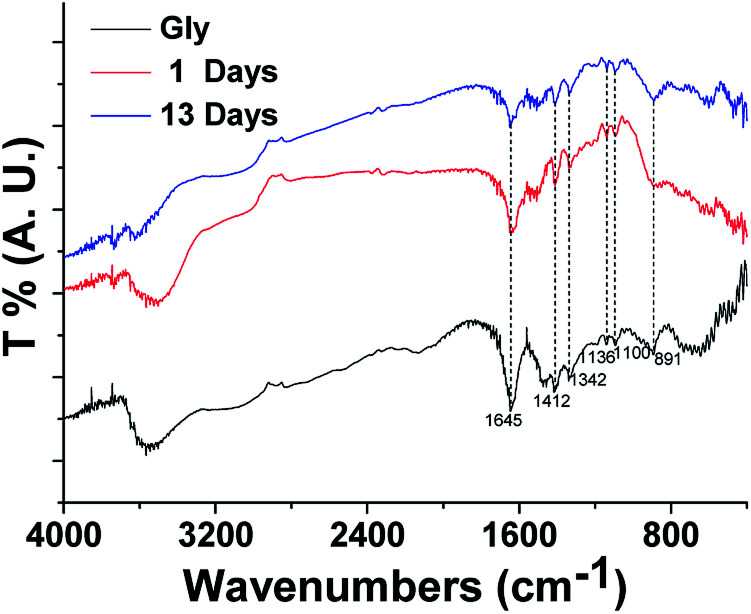
Measured FTIR spectra of glycine and aqueous BrC aerosol from HA-Gly mixtures.

The FTIR spectrum of glycine and HA-Gly mixtures after subtracting the baseline are shown in Fig. 6. In this region, there are some peaks that can be assigned to glycine: ∼891 cm^−1^ due to *ν*_s_(–CCN), ∼1100 cm^−1^ due to *ν*(–CN), ∼1136 cm^−1^ due to *ν*(–CN), ∼1342 cm^−1^ due to *ν*(–CH_2_–), ∼1412 cm^−1^ due to *ν*_s_(–COO^−^) and ∼1645 cm^−1^ due to *ν*(–NH_3_^−^).^46,47^ Glycine and HA-Gly mixtures used in our FTIR spectrum study have very similar size distributions. In other words, there is no significant difference in the measured FTIR spectrum between glycine and HA-Gly mixtures. It is difficult to discern absorption features in the FTIR spectra that could be attributable to organic chromophores. The experimental results of HA-Gly-AS mixtures are similar to that of HA-Gly mixtures. The FTIR spectrum of Gly-AS and HA-Gly-AS mixtures after subtracting the baseline are shown in Fig. S8.† In this region, there are some peaks that can be assigned to Gly-AS: ∼619 cm^−1^ due to *ν*(–COO^−^), ∼1114 cm^−1^ due to *ν*_3_(SO_4_^2−^), ∼1406 cm^−1^ due to *ν*_4_(NH_4_^+^) and ∼1635 cm^−1^ due to *ν*(–NH_3_^−^).^46–50^ Similarly, there is no significant difference in the measured FTIR spectrum between Gly-AS and HA-Gly-AS mixtures.

## Conclusion and discussion

The reacted solution shows a pronounced brownish discoloration that increases with reaction time and the measured UV/Vis absorption of HA-Gly and HA-Gly-AS mixtures are significantly enhanced in the 300–480 nm spectral range due to the formation of organic chromophores in the reaction. The results suggest that AS and Gly mixtures are more effective than only Gly on forming BrC *via* reactions with small aqueous carbonyl compound (HA) at room temperature.^51^ It is notable that the optical properties of HA-Gly and HA-Gly-AS mixtures should be dynamically changing. Moreover, our study provides fundamental information on the behavior of aqueous BrC by H_2_O_2_ oxidation of HA-Gly and HA-Gly-AS mixtures during photolysis processing.

Reactions between HA and Gly undergo rapid photobleaching *via* H_2_O_2_ oxidation photolysis. Light-absorbing compounds, which lose their ability to absorb the near-UV range radiation responsible for the slight browning color of BrC, can potentially rapidly photobleach during H_2_O_2_ oxidation photolysis. This indicates that the concentrations of aqueous BrC species will be higher during the night than in the daytime. In addition to the wavelength-dependent MAC values, more optical properties of two BrC samples were quantified in our study, namely, the effective rate *k* and the estimated effective half-life *τ* under typical atmospheric conditions. Aqueous BrC normally contain complex organic components; however, our measurements of the FTIR spectral properties show that Gly/Gly-AS and relevant aqueous BrCs have very similar size distributions. This is consistent with the previous conclusion that the reaction might have been generated from organic components, but the concentration is very low. Overall, our study provides the optical properties of the corresponding atmospheric aqueous BrC and the H_2_O_2_ oxidation photolysis process of the as-obtained BrC in detail, which may contribute to the understanding of the important effect of aqueous BrC on atmospheric chemistry and climate.

## Conflicts of interest

There are no conflicts to declare.

## Supplementary Material

RA-008-C8RA02019A-s001
