# Supplementary material for: Formation and photochemical investigation of brown carbon by hydroxyacetone reactions with glycine and ammonium sulfate
Source: RSC Adv. 2018 Jun 6;8(37):20719–25. doi: 10.1039/c8ra02019a (PMC9080858; doi:10.1039/c8ra02019a)
Supplement: RA-008-C8RA02019A-s001 [file RA-008-C8RA02019A-s001.pdf]

Electronic Supplementary Material (ESI) for RSC Advances.
This journal is © The Royal Society of Chemistry 2018
Electronic Supplementary Material (ESI) for RSC Advances.
This journal is © The Royal Society of Chemistry 2016

Electronic Supplementary Information

The Formation and Photochemical Investigation of Brown Carbon by Hydroxyacetone Reactions with Glycine and Ammonium Sulfate

Yan Gao and Yunhong Zhang*

School of Chemistry and Chemical Engineering, Beijing Institute of Technology, Beijing 100081, China.

School of materials and Chemical Engineering, Bengbu College, Bengbu 233030, China. E-mail:

Please do not adjust margins

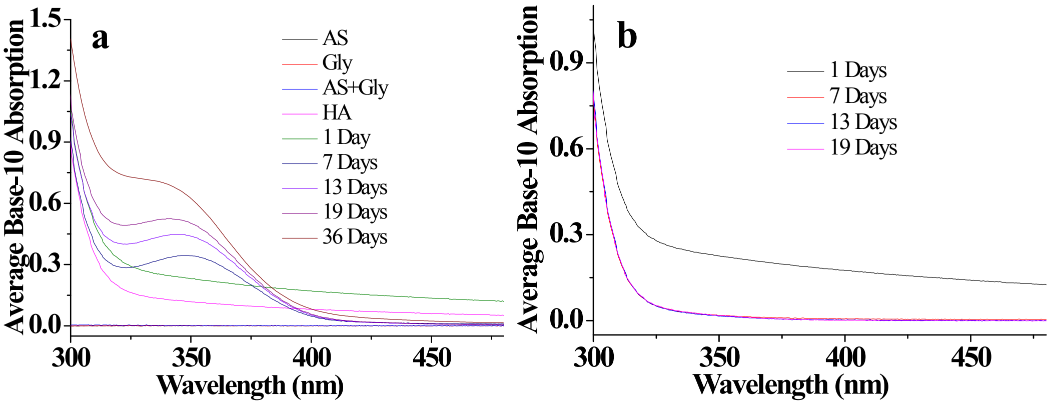

Fig. S1 UV/vis absorption spectra of HA-Gly-AS (a) and HA-AS mixtures (b) as a function of reaction time separately from 300-480 nm.

 This journal is © The Royal Society of Chemistry 20xx

Please do not adjust margins

Please do not adjust margins

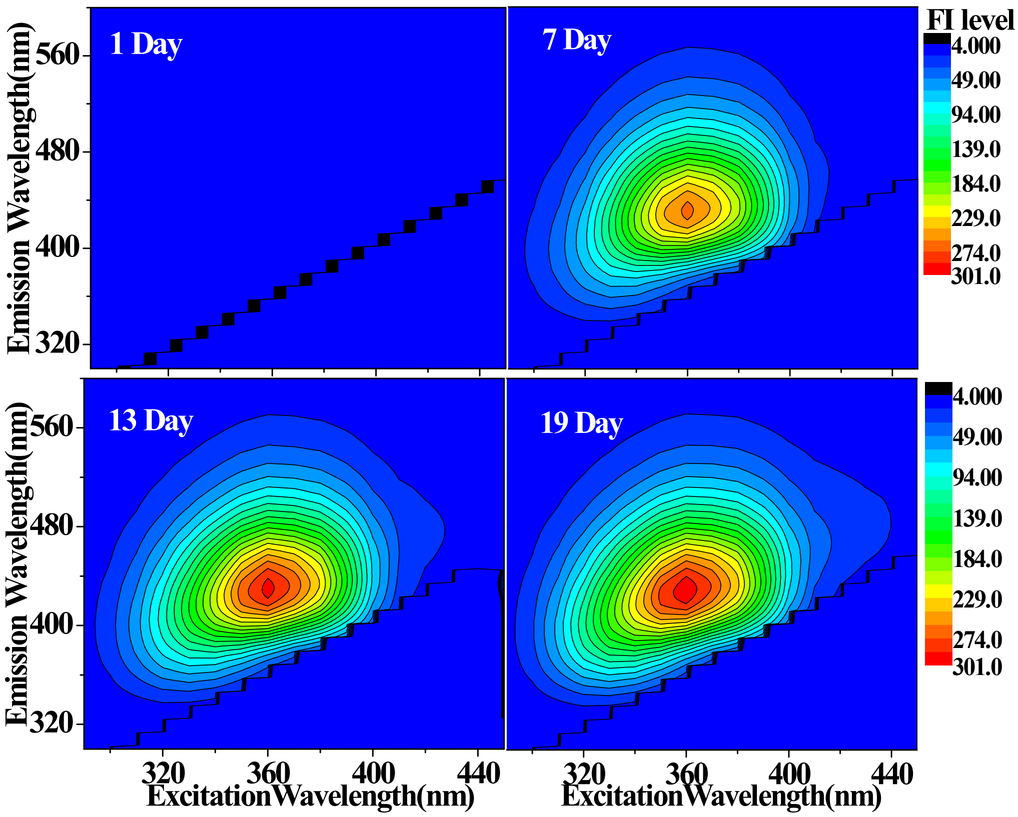

Fig. S2 EEM plots of HA-Gly-AS mixtures as a function of reaction time. The fluorescence intensity (FI level, color coded as shown on the right) increased as a function of reaction time.

This journal is © The Royal Society of Chemistry 20xx

Please do not adjust margins

Please do not adjust margins

solution	α (350 nm) cm^{-1}	I_{area}	QY %	AAE 400-480nm	FI	BIX
QS(0.058M)	0.00603	12269.4	51	-----	-----	-----
HA-Gly-AS(7 day)	0.23	23533.7	2.56	9.07	2.09	0.65
HA-Gly-AS(13 days)	0.34	26114.2	1.91	12.95	2.09	0.61
HA-Gly-AS(19 days)	0.44	27504.2	1.57	14.57	2.10	0.63
HA-Gly-AS(36 days) Before photolysis	0.62	27430.7	1.11	9.42	2.12	0.66
HA-Gly-AS(36 days) After photolysis	0.15	12856.4	2.15	7.86	2.35	0.86

Table S1 Measured base-10 absorption coefficients α correspond to 0.058 M QS solutions and 0.20 M HA-Gly-AS mixtures. The integrated emission intensities I_{area} correspond to the 350 nm excitation from 362 to 600 nm.

This journal is © The Royal Society of Chemistry 20xx

Please do not adjust margins

Please do not adjust margins

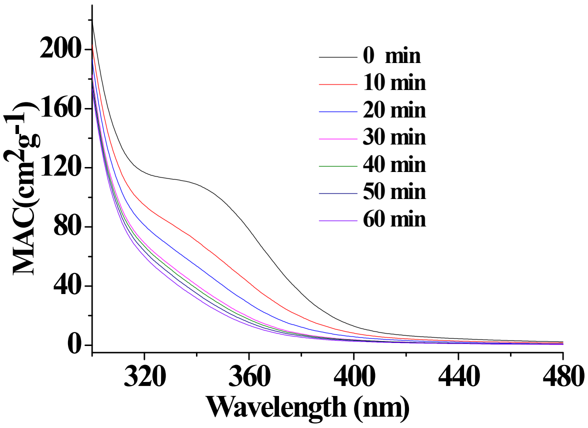

Fig. S3 UV/vis absorption spectra recorded during photolysis of HA-Gly-AS mixtures on the 300-480 nm range.

This journal is © The Royal Society of Chemistry 20xx

Please do not adjust margins

Please do not adjust margins

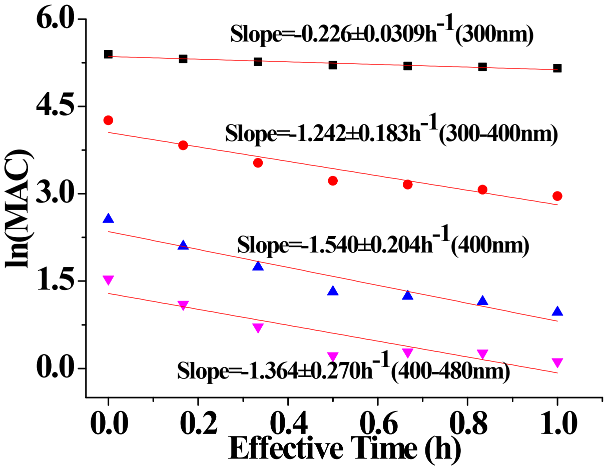

Fig. S4 Decay of absorbance during irradiation of HA-Gly aqueous mixtures.

This journal is © The Royal Society of Chemistry 20xx

Please do not adjust margins

Please do not adjust margins

Wavelength(nm)	300	near-UV	400	visible
k	0.226	1.242	1.54	1.364
τ	3.067	0.558	0.450	0.508

Table 2 Effective rate constant *k* and effective half-life τ (in h) for the disappearance of absorbance at different wavelengths in HA-Gly-AS mixtures.

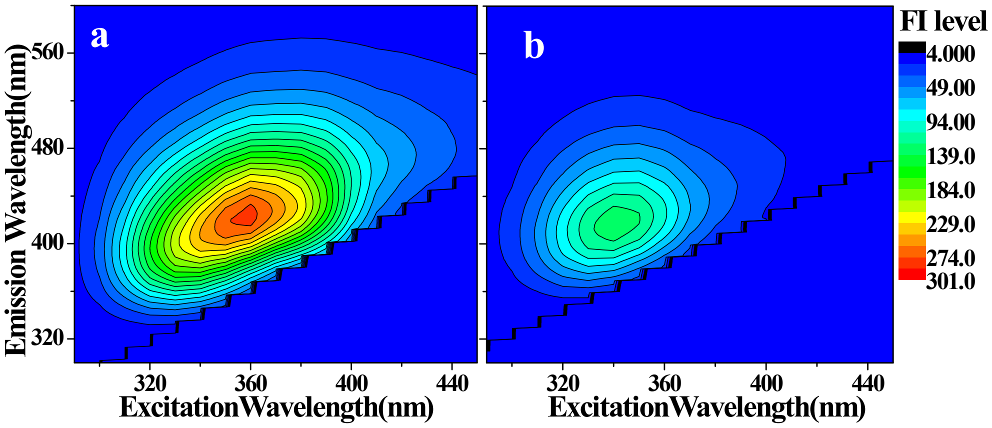

Fig. S5 EEM spectrum recorded (a) before and (b) after photolysis of HA-Gly-AS mixtures.

Please do not adjust margins

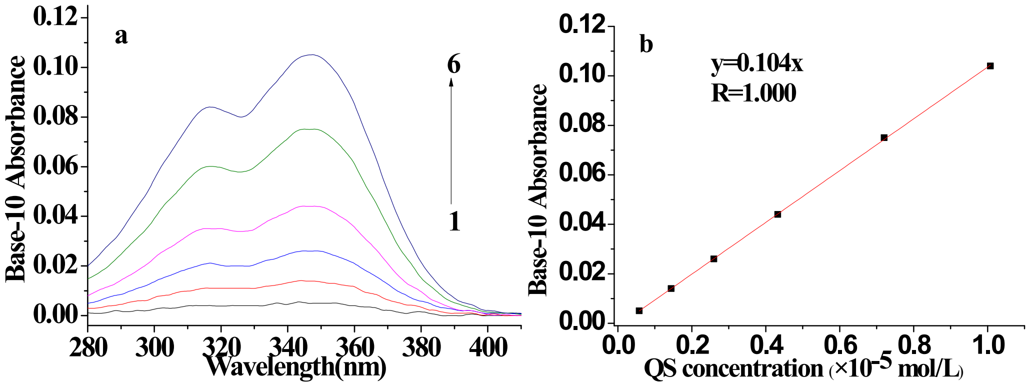

Fig. S6 (a) Absorption spectra of solutions of QS ($C_{40}H_{48}N_4O_4$▪H_2SO_4▪$2H_2O$) recorded at molar concentrations of 0.058, 0.144, 0.260, 0.433, 0.720, 1.008 $\times 10^{-5}$ M（1-6）. These mixtures also pertained to the fluorescence measurements in Fig. S7(a). Base-10 absorbance of QS was measured for the quantum yield measurements. **Fig. S6(b)** Calibration of the measured base-10 absorbance vs. QS molar concentration in M.

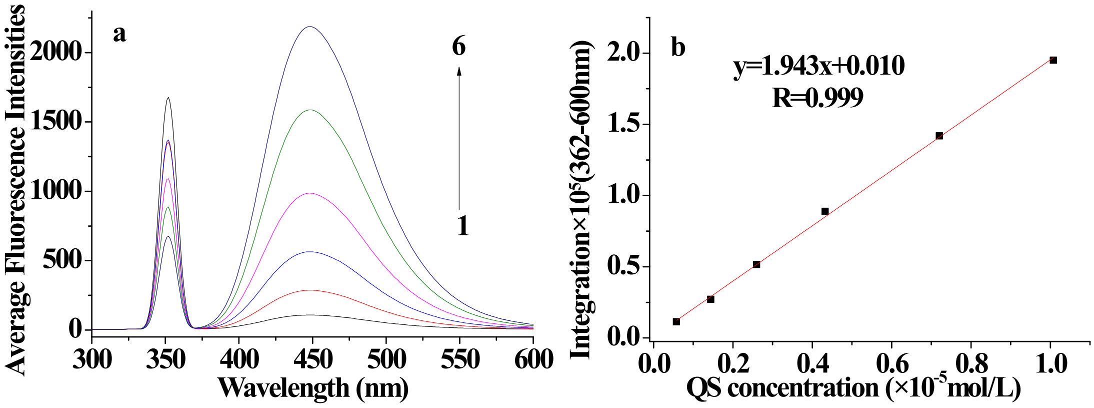

Fig. S7(a) Fluorescence measurements of QS solutions recorded at molar concentrations of 0.058, 0.144, 0.260, 0.433, 0.720, 1.008 $\times 10^{-5}$ M（1-6）. Average fluorescence intensities of QS were measured for the quantum yield measurements. **Fig. S7(b)** Calibration of the integral area of fluorescence intensities vs. QS molar concentration in M.

This journal is © The Royal Society of Chemistry 20xx

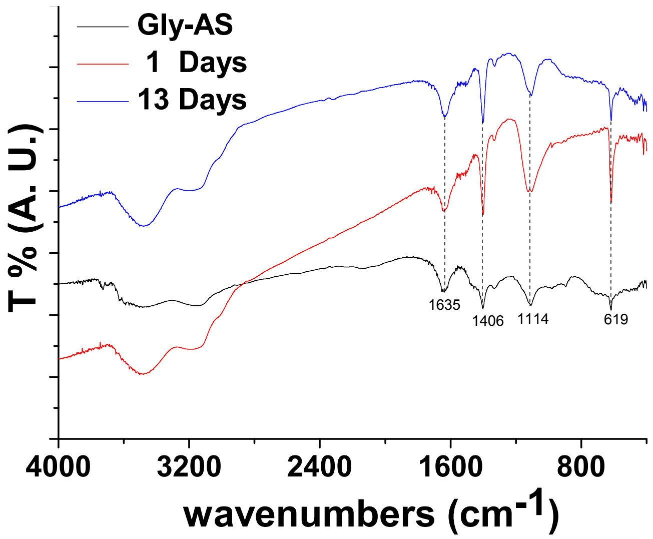

Fig. S8 Measured FTIR spectra of glycine and aqueous BrC aerosol from HA-Gly-AS mixtures.
